# Differentiation between blood and iodine in a bovine brain—Initial experience with Spectral Photon-Counting Computed Tomography (SPCCT)

**DOI:** 10.1371/journal.pone.0212679

**Published:** 2019-02-25

**Authors:** Isabelle Riederer, Salim Si-Mohamed, Sebastian Ehn, Daniel Bar-Ness, Peter B. Noël, Alexander A. Fingerle, Franz Pfeiffer, Ernst J. Rummeny, Philippe Douek, Daniela Pfeiffer

**Affiliations:** 1 Department of Diagnostic and Interventional Radiology, Technical University of Munich, Munich, Germany; 2 Department of Diagnostic and Interventional Neuroradiology, Technical University of Munich, Munich, Germany; 3 Department of Interventional Radiology and Cardio-vascular and Thoracic Diagnostic Imaging, Louis Pradel University Hospital, Bron, France; 4 University Claude Bernard Lyon 1, CREATIS, CNRS UMR 5220, INSERM U1206, INSA-Lyon, France; 5 Chair of Biomedical Physics & Munich School of BioEngineering, Technical University of Munich, Garching, Germany; University of Notre Dame, UNITED STATES

## Abstract

**Objectives:**

To evaluate the accuracy of Spectral Photon-Counting Computed Tomography (SPCCT) in the quantification of iodine concentrations and its potential for the differentiation between blood and iodine.

**Methods:**

Tubes with blood and a concentration series of iodine were scanned with a preclinical SPCCT system (both in vitro and in an ex vivo bovine brain tissue sample). Iodine density maps (IDM) and virtual non-contrast (VNC) images were generated using the multi-bin spectral information to perform material decomposition. Region-of-interest (ROI) analysis was performed within the tubes to quantitatively determine the absolute content of iodine (mg/ml).

**Results:**

In conventional CT images, ROI analysis showed similar Hounsfield Unit (HU) values for the tubes with blood and iodine (59.9 ± 1.8 versus 59.2 ± 1.5). Iodine density maps enabled clear differentiation between blood and iodine *in vitro*, as well as in the bovine brain model. Quantitative measurements of the different iodine concentrations matched well with those of actual known concentrations even for very small iodine concentrations with values below 1mg/ml (RMSE = 0.19).

**Conclusions:**

SPCCT providing iodine maps and virtual non-contrast images allows material decomposition, differentiation between blood and iodine *in vitro* and *ex vivo* in a bovine brain model and reliably quantifies the iodine concentration.

## Introduction

Unenhanced head computed tomography (CT) is commonly performed within the first day after mechanical thrombectomy in ischemic stroke patients to assess for early complications such as haemorrhage[1]. However, the differentiation between blood and extravasation of iodine-based contrast material due to a disruptured blood brain barrier is often difficult because of the similar Hounsfield Unit (HU) values. Therefore, follow-up examinations might be necessary that then delay final diagnosis and therapy management. Thus, early differentiation is important to provide best possible treatment, especially for therapy adjustment regarding anticoagulation therapy or treatment with antiplatelet agents.

In the last years, spectral CT imaging methods [2] have increasingly been used in research and clinical practice to simultaneously evaluate anatomy and tissue composition. This is possible because x-ray attenuation is energy- and material-dependent. Using a dedicated material decomposition scheme prior to image reconstruction, virtual non-contrast images (VNC) and iodine density maps (IDM) can be reconstructed to differentiate between blood and iodinated contrast material. Some studies have already shown that differentiation between haemorrhage and extravasation of iodine-based contrast material due to a disrupted blood brain barrier in ischemic stroke patients after mechanical thrombectomy is possible with dual-energy CT (DECT) [3–5]. Recently, we could show a high accuracy in iodine quantification using dual-layer CT (DLCT) that represents a technology in between traditional DECT and detector-based Spectral CT [6]. The concept of dual-energy CT is based on an examination using two different acceleration voltages, either from two different x-ray sources[7] or from one x-ray source switching between two different kV settings[8–10].

Alternatively, a detector-based approach, Spectral Photon-Counting CT (SPCCT), can be used where x-ray photons are individually counted and spectrally binned by analyzing the pulse heights generated in a semi-conductor detection layer [11–13, 9]. This concept allows to incorporate a multiple (more than two) energy bins for energy-selective data acquisition. A recently published review [14] summarized that photon-counting CT is a promising technique that might extend and improve the clinical use of CT in the future. Photon-counting CTs can lower image noise, increase spatial resolution, and reduce radiation doses by at least 30%–40% [14]. Another study conformed the potential for high resolution and further concluded that high accuracy for iodine quantification and improved contrast to noise ratio is feasible with SPCCT [15]. Furthermore, and by using k-edge imaging [16], it is possible to differentiate between gadolinium-based and non-ionic iodine-based contrast material. This has been demonstrated in a colon phantom [17], a heart model in animals *in vivo* [18], between targeted gold nanoparticles, iodine-based contrast agent and calcium phosphat [19], or even between three different contrast agents (bismuth, gadolinium-based and iodine-based contrast material) in an abdomen in animals *in vivo* [20].

Furthermore, pilot studies have already demonstrated the possibility of using SPCCT for diagnostics of the abdomen [21] and vascular imaging of head and neck [22] in humans with promising results. Thus, it seems likely that clinical CT imaging will benefit from SPCCT in the future after further development of scanners with enlarging the FOV size to clinical relevant sizes.

In this study, we investigated the potential of SPCCT for the differentiation between blood and iodine *in vitro* and in an *ex vivo* bovine brain model and for the quantification of different iodine concentrations.

## Material and methods

### Scan specimens

**Fig 1:**
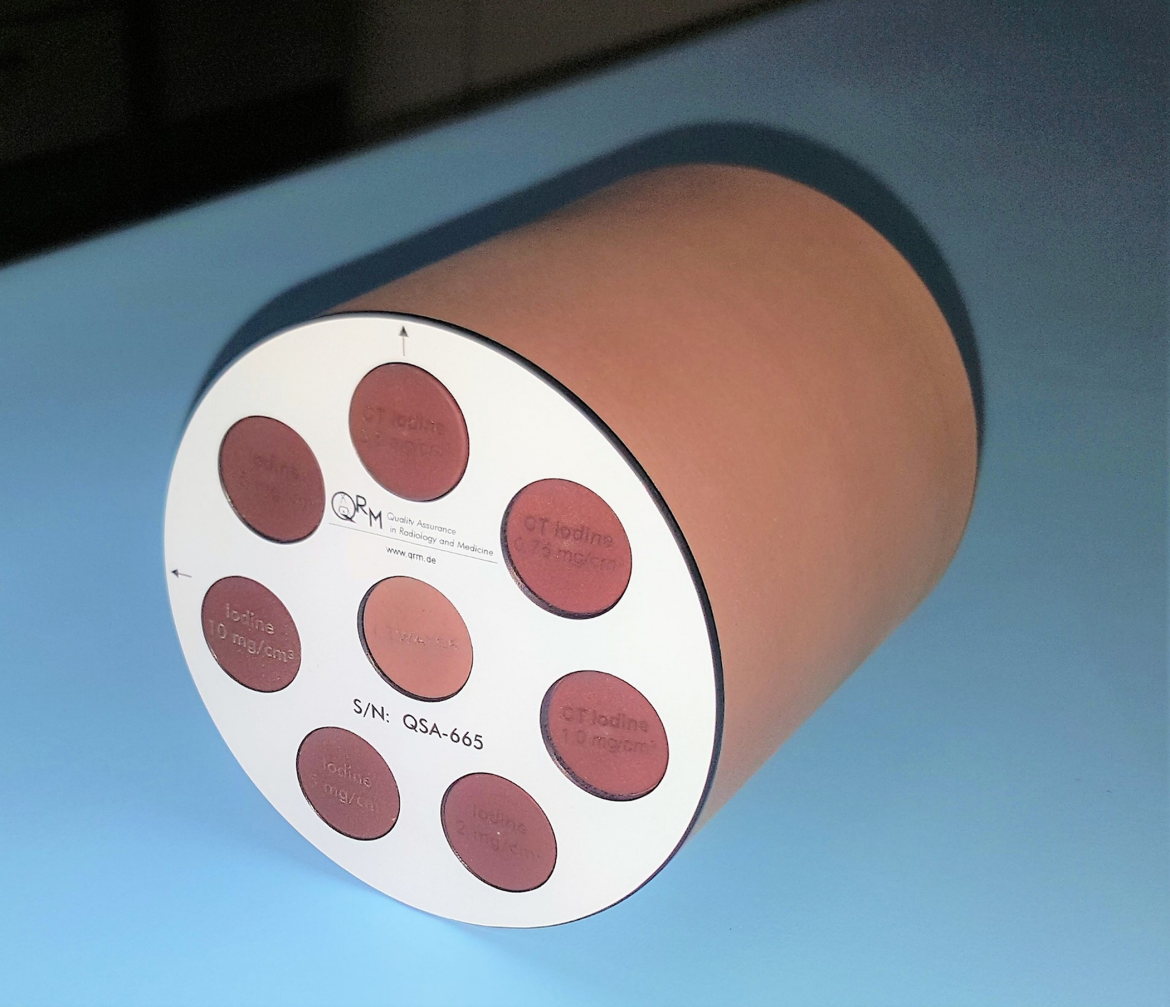
Phantom model with inserts with different concentrations of iodine (QRM GmbH, Forchheim, Germany).

First, a *quantitative phantom experiment* was performed using a blood sample taken from a volunteer, and inserts with different concentrations of iodine (0.5 mg/ml; 0.75 mg/ml; 1 mg/ml; 2 mg/ml; 5 mg/ml and 10 mg/ml CTIodine^®^; QRM GmbH, Forchheim, Germany) embedded into a solid cylinder of water-equivalent material and 10 cm diameter (**Fig 1**). Scans were repeated four times and each scan included three different scan positions. The volunteer gave written informed consent and the study was approved by the local ethics committee (Ethikkommission der Fakultät für Medizin der Technischen Universität München) Number: 97/18s.

Second, a *simulation model experiment* was performed with tubes of (a) blood prepared as explained above and (b) iodine-based contrast material (2 mg/ml; Bracco, Milan, Italy), which were positioned within fresh *bovine brain tissue* (commercially available from a butcher). Here, a low iodine concentration (2 mg/ml) was chosen to obtain similar HU values compared to pure blood in the conventional CT images.

### Spectral photon-counting CT

All experiments were performed with a five bins SPCCT system (Philips Healthcare, Haifa, Israel) to obtain spectral and conventional data. SPCCT is based on a semiconductor detector technology operated in single photon-counting mode with energy discrimination using 5 bins set as 30, 51, 64, 72, and 85 keV. The in-plane field of view was 168 mm, with a z-coverage in the scanner isocenter of 2.5 mm. Axial scans over 360° were obtained with a tube current of 100 mA, a tube voltage of 120 kVp, a scanner rotation time of 1 second, and 2400 projections per rotation[23].

### Material decomposition and quantitative measurements

Multi-bin photon-counting data were pre-processed, and a conventional CT image was derived from the summed information contained in all energy bins. In addition, after pileup correction, the multi-bin counting data were used to perform a maximum likelihood-based material decomposition into a water and iodine material basis[11, 12] in projection space. Iodine was decomposed and quantified from the blood/iron background. Pile-up is corrected by a look-up table, which relates actual photon flux to the one counted in the different energy bins, containing pile-up. The material-decomposed projections have been reconstructed using FBP and no post processing was done to further reduce image noise on FBP images. All images were reconstructed on a voxel grid of 0.39 × 0.39 × 0.25 mm^3^. The iodine and virtual non-contrast images were averaged to a slice thickness of 1 mm after CT reconstruction.

### Region of interest analysis

First, reference scans were performed to calibrate the following measurements. Then, measurements of all tubes and inserts, as described above, were performed. Here, region-of-interest (ROI) analysis was performed within the tubes to quantitatively determine the absolute content of iodine concentration using ImageJ (National Institutes of Health (NIH), United States[24]). A circular ROI of 120 mm^2^ was drawn in the center of the probes to perform measurements.

### Statistical analysis

The iodine concentration measured with SPCCT was correlated to the true iodine concentration by Pearson correlation and student´s t-test. Root mean square error (RMSE) was calculated. Additionally, Bland-Altman analysis was performed to determine the agreement between measured and true iodine concentrations.

## Results

### In vitro experiments

**Fig 2:**
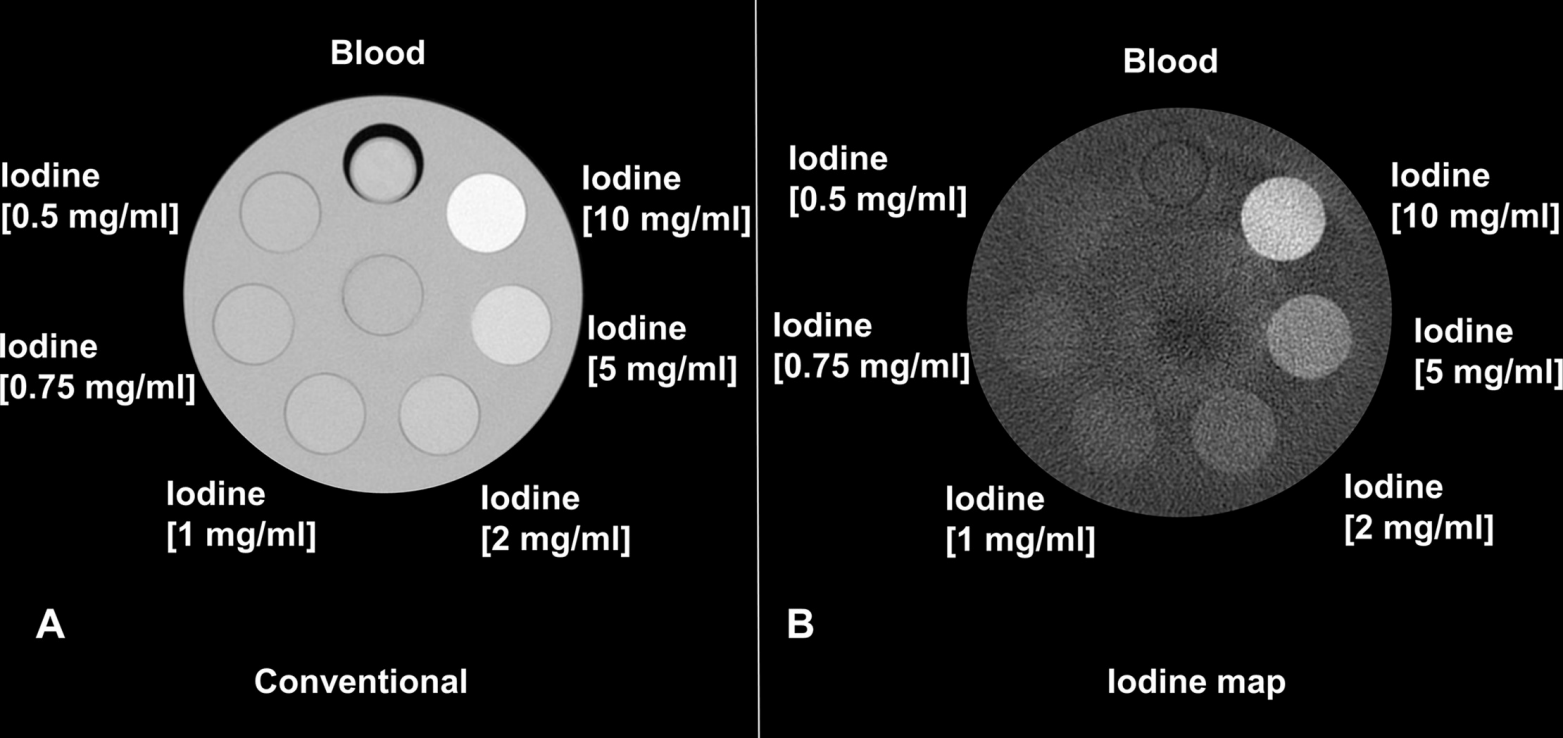
Phantom sample with blood and inserts with varying iodine concentrations as indicated in the image. (A) Conventional CT, where blood cannot be differentiated from the lower iodine concentrations. (B) Iodine map, where lower iodine concentrations are discernible from the blood sample. Visually, already the smallest measured iodine concentration of 0.5 mg/ml can be discriminated from blood.

**Fig 3:**
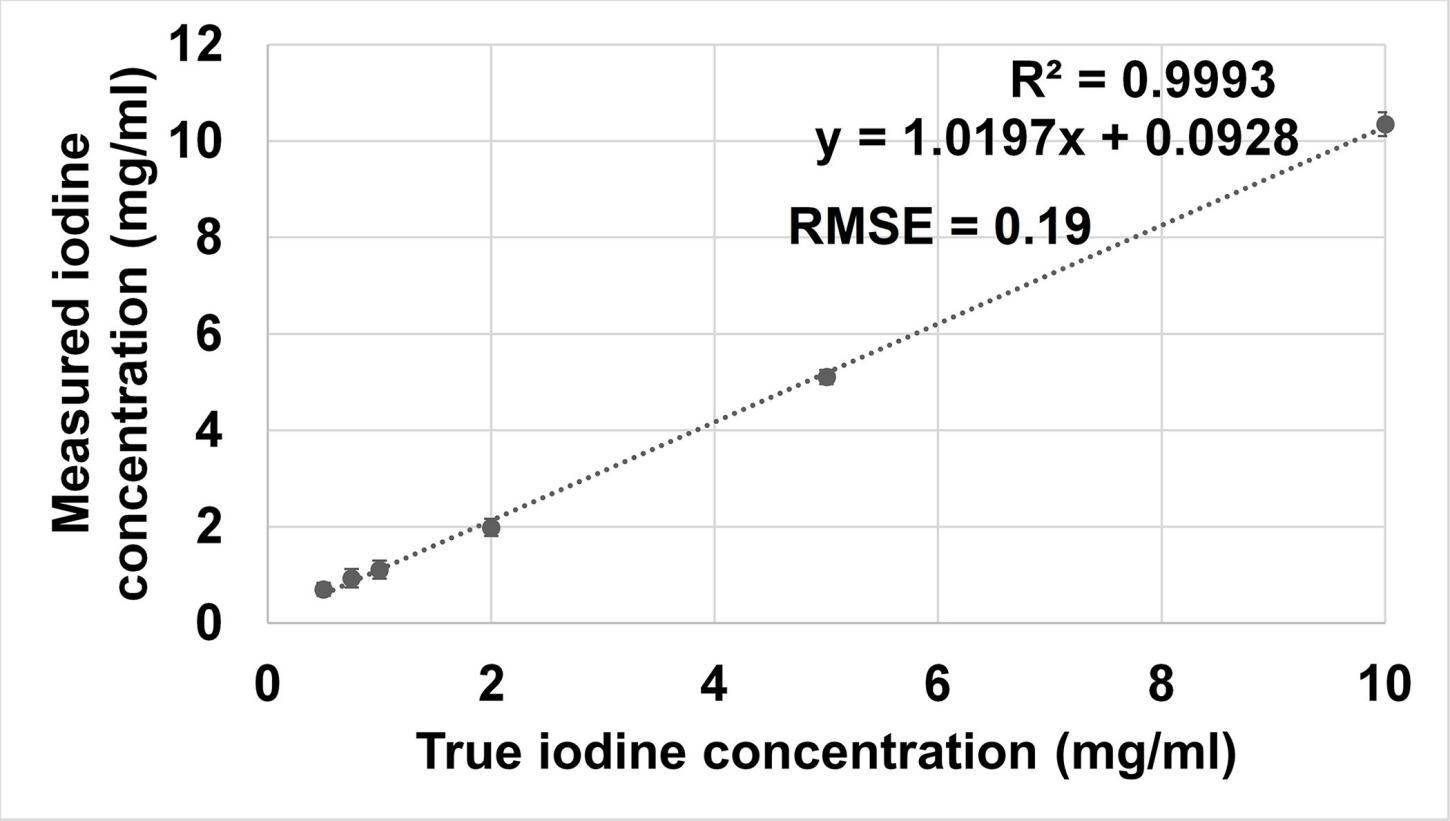
Scatter plot showing correlation between iodine concentration measured with SPCCT and true iodine concentration contained in the phantom (R^2^ = 0.9993, p < 0.03).

**Fig 4:**
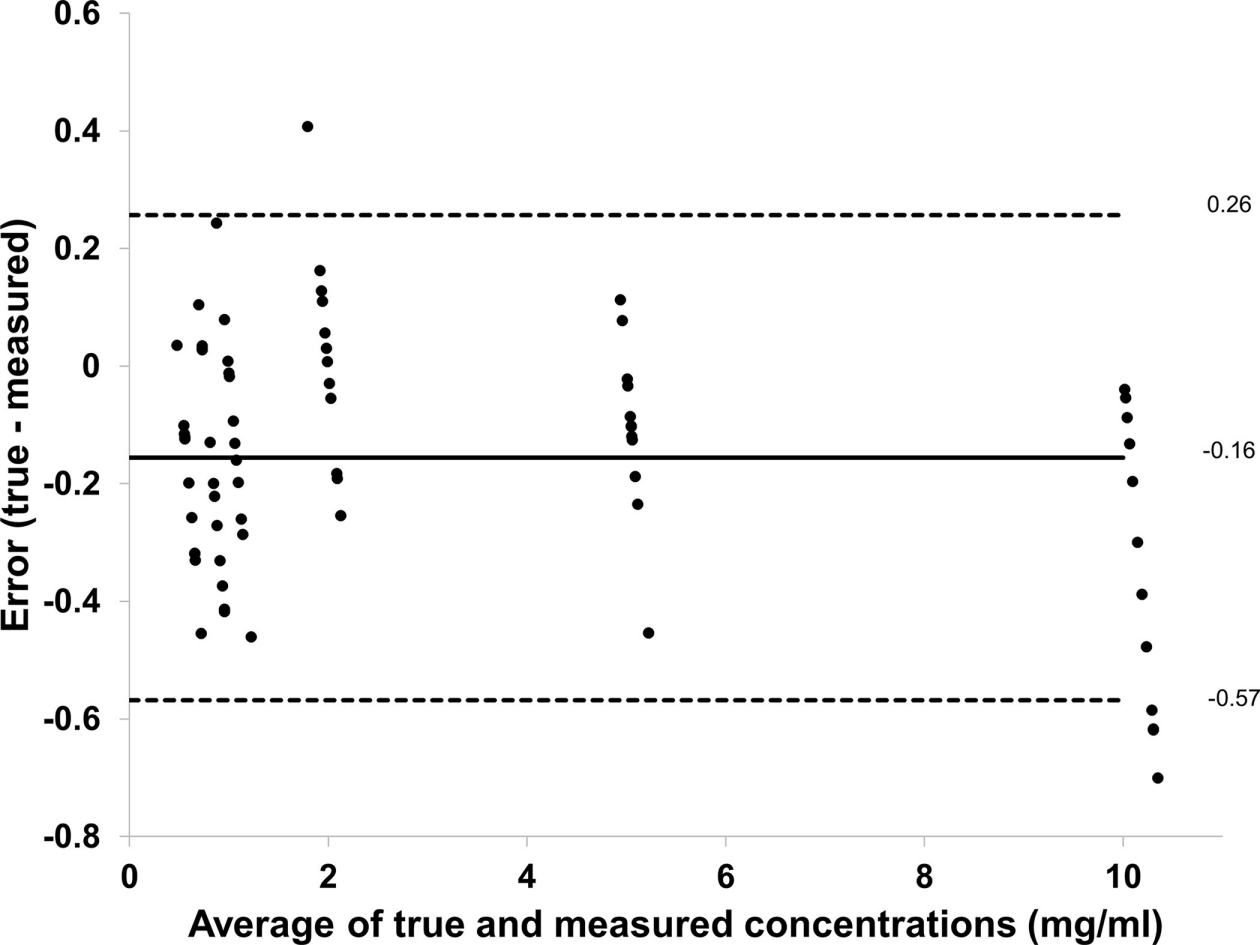
Bland-Altman plot showing difference between true and measured iodine concentrations versus average of true and measured iodine concentrations. The black line represents the bias and the dashed lines represent upper and lower limits of the mean (confidence limits ± 1.96).

Iodine density maps enabled clear differentiation between blood and iodine *in vitro* (**Fig 2**). In the obtained IDM, already the smallest used iodine concentration (0.5 mg/ml) could be visually discriminated from the blood sample. Furthermore, spectral photon-counting CT enabled quantitative measurements of different iodine concentrations. The quantitative measurements of the inserts with different iodine concentrations matched well with those of actual known mixtures (measured: 0.70 ± 0.14 mg/ml, actual: 0.5 mg/ml; measured: 0.93 ± 0.19 mg/ml, actual: 0.75 mg/ml; measured: 1.11 ± 0.19 mg/ml, actual: 1 mg/ml; measured: 1.98 ± 0.18 mg/ml, actual: 2 mg/ml; measured: 5.11 ± 0.15 mg/ml, actual: 5 mg/ml; measured: 10.35 ± 0.24 mg/ml, actual: 10 mg/ml; RMSE = 0.19; Pearson´s correlation = 0.998). **Fig 3** shows a scatter plot displaying excellent correlation between measured iodine concentration and true iodine concentration (R^2^ = 0.9993, p < 0.03). A Bland-Altman plot showing differences between true and measured iodine concentrations versus the average of true and measured iodine concentrations is shown in **Fig 4**. Nearly all measurements (94%, 68/72) were located within the range of the confidence limits.

### Biological phantom model

**Fig 5:**
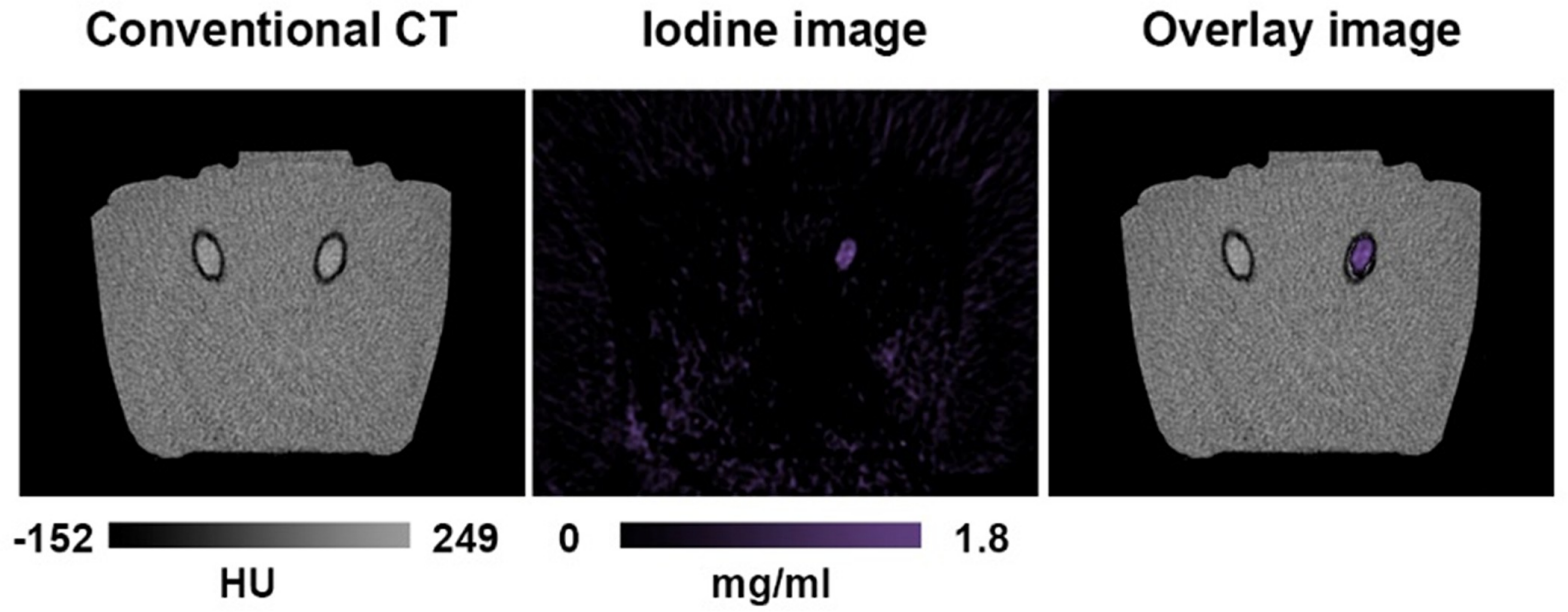
Tubes with blood or iodine-based contrast material positioned in bovine brain tissue (*ex vivo*). In conventional CT (left) the content of the tubes cannot be differentiated due to similar HU values. In the iodine (middle) and overlay (right) image the tubes are clearly distinguishable due to the decomposition algorithm.

In the conventional CT images, the ROI analysis showed similar HU density values for the tubes with blood and iodine (blood: 59.9 ± 1.8 versus iodine: 59.2 ± 1.5), thus differentiation between both materials was not possible in conventional CT images. Iodine maps enabled clear differentiation between blood and iodine in the bovine brain (**Fig 5**). Quantitative measurements showed an iodine concentration of 2.1 ± 0.8 mg/ml (prepared concentration was 2 mg/ml).

## Discussion

In this study, we demonstrated that SPCCT enables discrimination between blood and iodine-based contrast material *in vitro* and in an *ex vivo* bovine brain model. Furthermore, SPCCT allows reliable quantification of different iodine concentrations *in vitro*.

Hyperdense areas in the brain parenchyma are a frequent finding (25–84%) [25, 26] in control CTs of the head after interventional thrombectomy and are significantly more often associated with haemorrhagic transformation of the infarction[25]. One study concluded that hyperdense lesions seem not to be a predictor of poor outcome[25]. However, the risk of deterioration seems to be significantly increased in patients with cerebellar infarction and hemorrhagic conversion[27]. Therefore, differentiation between blood and extravasation of iodinated contrast material is highly relevant.

Compared to other studies that quantified iodine in dual-energy CTs[28, 29], we extended the range of iodine quantification towards very low levels in the range of 0.5–2.0 mg/ml. In our study, we could show that there is a strong correlation between the measured and the true iodine concentrations using SPCCT. Quantification of iodine concentrations can be relevant to detect lesions and characterize tissue composition[30], for example when distinguishing pulmonary artery sarcoma from pulmonary thromboembolism[31] or clear cell from papillary renal cell carcinoma[32].

A limitation of our study is the absence of any real *in vivo* patient data; however, this is not possible at this time as the FOV of the used pre-clinical SPCCT scanner is too small for adult human patients–however a FOV typical of clinical scanners could be achieved via development of the detector array. Furthermore, surrounding bone-like tissue with corresponding beam hardening is missing that could influence the accuracy of iodine quantification. In the future, it will be necessary to translate our results to a clinically relevant field-of-view in an *in vivo (animal)* model. In addition, another study has to be performed with multiple concentrations of iodine in this or a similar biological model to evaluate the quantitative accuracy.

## Conclusions

In conclusion, Spectral Photon-Counting CT provides iodine density maps and allows for material decomposition and differentiation between blood and iodine *in vitro* and within an *ex vivo* bovine brain model. Furthermore, reliable quantification of different iodine concentrations is feasible *in vitro*. The introduction of such a system into the clinical field may improve diagnostic imaging.

## Abbreviations

SPCCT: Spectral Photon-Counting Computed Tomography
IDM: Iodine density maps
VNC: virtual non-contrast
RMSE: root mean square error
